# Clinical assessment of prophylactic chemotherapy in treating with hydatidiform mole: A protocol for systematic review and meta-analysis

**DOI:** 10.1097/MD.0000000000026341

**Published:** 2021-06-18

**Authors:** Feng Xu, Yan-Li Zheng, Xiao-Yan Lu, Hai-Feng Qiao, Ying Wang

**Affiliations:** Department of Obstetrics and Gynecology, the First People's Hospital of Nantong, Nantong, Jiangsu, China.

**Keywords:** efficacy, hydatidiform mole, meta, prophylactic chemotherapy, safety

## Abstract

**Background:**

Hydatidiform mole (HM) is more common as molar pregnancy. It is a disease classified under the category of gestational trophoblastic diseases, which could metastasize after originating in the placenta. A majority of females suffering from molar pregnancies are curable by evacuating retained products of conception and the patient's fertility is preserved. In some cases, the growth perseveres and leads to gestational trophoblastic neoplasia, which is an extremely malicious condition that needs chemo-based treatment. There is a possibility to lessen the risk of gestational trophoblastic disease in females with HM through the administration of prophylactic chemo. Yet, there is controversy regarding prophylactic chemotherapy administered pre-or-post removal of HM to curtail the malignant sequelae. Therefore, we will conduct this research to assess both the efficacy as well as security of using prophylactic chemotherapy to treat HM.

**Methods:**

In the preliminary review, the authors will search for randomized controlled trials involving prophylactic chemotherapy to treat HM. The literature search is carried out in the following electronic databases from their inception to May 2021: Chinese National Knowledge Infrastructure, Chinese BioMedical Literature, and WanFang database are the three Chinese language databases. Web of Science, PubMed, Cochrane Library, and EMBASE are the four English language databases. The authors will also perform a manual search through the bibliographies in related literature to find extra articles and ongoing studies. Two independent authors will assess the literature according to an inclusion criteria, use a specialized data collection table to extract data, and use the Cochrane ‘Risk of bias’ tool for evaluating any possible bias risk in the selected articles. Data synthesis and statistical operations are completed with the RevMan software (v. 5.3).

**Results:**

The present systematic analysis provides a rationalized synthesis of existing evidence related to the use of prophylactic chemotherapy in the treatment of HM.

**Conclusion:**

Our findings will summarize the current evidences for prophylactic chemotherapy in the treatment of HM.

**Ethics and dissemination:**

An ethics approval is nonrequired because pre published results will be used.

**Registration number:**

DOI 10.17605/OSF.IO/6QV52 (https://osf.io/6qv52/)

## 1 Introduction

Gestational trophoblastic disease (GTN) refers to a condition characterized by a relentless autonomous excessive growth of embryonic chorionic tissue or trophoblast.^[1,2]^ Hydatidiform mole (HM), commonly referred to as molar pregnancy is classified under the category of GTN, which has a metastasizing probability after originating in the placenta. HM is categorized as a whole and part mole according to the gross morphology, histopathology, and karyotype, and is generally regarded as a non-invasive type of GTN.^[3–6]^ Even though HM is regarded as benign, they are pre-malignant and has the likelihood to turn invasive and malignant. Vaginal bleeding is the most common symptom of HM. Accompanying symptoms are more common in complete mole, including theca lutein ovarian cysts, hyperthyroidism, and excessive uterine enlargement. Still, such conditions are far less prevalent because frequent ultrasound scans lead to early diagnosis.^[7]^ The conception's retained products are evacuated in females who desire to preserve their fertility, preferably by suction curettage, to completely eradicate all trophoblastic tissue.^[7]^ A majority of the cases can be treated in such a manner. However, in some females, HM remains prevalent and advances to a malignant nature that requires chemo-based treatment.^[8,9]^

GTN is a condition that is extremely chemo-sensitive, and different chemotherapeutic agents have achieved decent rates of cure. According to the level of GTN, it is either classified as low- or high-risk GTN. Over the recent years, the therapeutic strategy used to treat low-risk GTN has remained largely unchanged. All females with “low-risk" GTN and nearly 85% of females with “high-risk" GTN are treated and healed using a single chemo agent or a combination of chemo-based treatments.^[7,10,11]^ Dactinomycin and methotrexate are widely regarded to be fairly safe agents as the first-line of chemo for GTN, either singularly or combined with other chemotherapeutic agents.^[12,13]^ The first usage of prophylactic chemo for females with HM took place in 1966.^[14]^ Until now, no previous research has explored the effectiveness and safety of prophylactic chemotherapy in the treatment of HM. Therefore, in the present research, we will assess the efficacy and security of using prophylactic chemotherapy to treat HM.

## 2 Objective

The aim of this meta-analysis is to systematically investigate the effectiveness and safeness associated with the use of prophylactic chemotherapy to treat HM.

## 3 Methods

### 3.1 Study registration

This study will be conducted according to the guidelines outlined in the Preferred Reporting Items for Systematic Reviews and Meta-Analyses Protocols (PRISMA-P). This study is registered in the OSF (https://osf.io/) with DOI 10.17605/OSF.IO/6QV52.

### 3.2 Criteria for considering studies for this study

#### 3.2.1 Types of studies

The authors will consider all randomized controlled trials as well previously published articles that have evaluated the use of prophylactic chemotherapy to treat HM.

#### 3.2.2 Types of participants

All females diagnosed with HM are considered as participants. No restrictions will be applied in terms of age, country, and ethnicity.

#### 3.2.3 Types of interventions/comparisons

All participants in the experimental group must have received prophylactic chemotherapy. Those in the control group must have been treated with placebo, analgesic drugs, or no treatment.

#### 3.2.4 Types of outcomes

The primary outcomes are incidence of GTN, which includes invasive mole, choriocarcinoma, and both epithelioid trophoblastic and placental site tumors. The secondary outcomes include time to diagnose GTN, occurrence of successive pregnancies, overall survival, drug toxicity, and standard of life.

### 3.3 Search methods for identification of studies

In the preliminary review, the authors will search for randomized controlled trials involving prophylactic chemotherapy to treat HM. The literature search is carried out in the following electronic databases from their inception to May 2021: Chinese National Knowledge Infrastructure, Chinese BioMedical Literature, and WanFang database are the three Chinese language databases. Web of Science, PubMed, Cochrane Library, and EMBASE are the 4 English language databases. The authors will also perform a manual search through the bibliographies in related literature to find extra articles and ongoing research. The following search terms will be used individually or in combinations: “hydatidiform mole," “prophylactic chemotherapy," AND “randomized controlled trial."

### 3.4 Data collection and analysis

#### 3.4.1 Selection of studies

**Figure 1:**
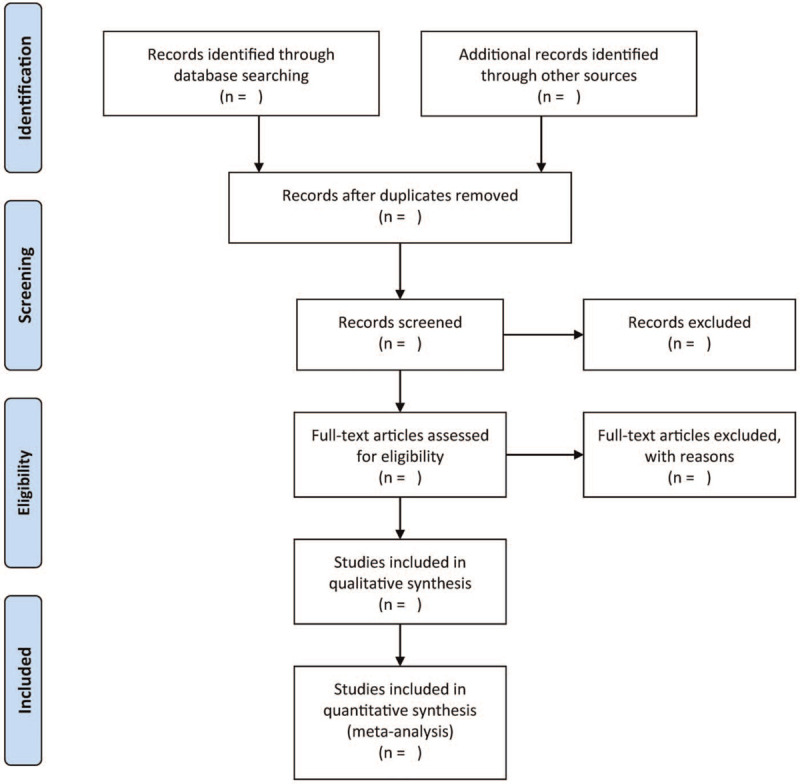
The work flowchart.

Two independent authors will review the retrieved studies. Briefly, they will exclude duplicate articles and studies that do not match the inclusion criteria by reading titles/abstracts. Afterwards, the full-texts are scrutinized, having met the above criteria, the studies are selected for further screening and will be included in the meta-analysis. A third author will evaluate any discrepancies if necessary. Figure 1 is a summary of the process that will be used for selecting studies.

#### 3.4.2 Data extraction and management

A pair of autonomous authors will use a specially designed data collection table to extract data and summarize details from selected studies. All the data that are extracted shall be reviewed for accuracy and completeness. The extracted information will include study characteristics (study type, publication date, country, and ethnicity), design (method, intervention chemotherapy regimen, and dosage), and outcome measures. A third author will evaluate any discrepancies if necessary.

#### 3.4.3 Assessment of risk of bias

The authors will employ the Cochrane Collaboration tool to assess the bias risk in the studies selected.^[15]^ A third author will evaluate any discrepancies if necessary.

#### 3.4.4 Measures of treatment effect

Dichotomous outcomes are presented as relative risk and 95% confidence intervals, whereas continuous outcomes are presented as mean differences or standardized mean differences and 95% confidence intervals.

#### 3.4.5 Assessment of heterogeneity

The authors are planning to use the *χ*^2^ test and *I*^2^ statistic to determine statistical heterogeneity. If the P < .1, or *I*^2^ > 50%, the random-effect model will be employed for analysis. Else, if the *P* > .1 or the *I*^2^ < 50%, then the fixed-effect model will be employed.

#### 3.4.6 Assessment of reporting biases

If applicable, the potential bias in publication will be assessed using Funnel plots. Meanwhile, asymmetry on the funnel plot will also be checked using Egger test.

#### 3.4.7 Sensitivity analysis

The authors are also planning to conduct a sensitivity analysis to assess the robustness of the results.

## 4 Discussion

Scholars predict that prophylactic chemotherapy has a significant role in the treatment of HM patients. Yet, up to date, published studies related to the use of prophylactic chemotherapy on HM have been mostly theoretical. Considering the growing body of literature on prophylactic chemotherapy for HM, it is useful to conduct a systematic meta-analysis to determine the efficacy and safety of using prophylactic chemotherapy to treat HM patients. Therefore, this study will summarize the existing body of knowledge related to the efficacy and safeness of prophylactic chemotherapy for treating HM. Therefore, the outcomes of the study will provide dependable references for practitioners and patients when treating HM patients with prophylactic chemotherapy. Most importantly, the results could provide policymakers with fresh insights to an alternative form of prophylactic chemotherapy therapy.

## Author contributions

**Conceptualization:** Feng Xu.

**Data curation:** Yan-Li Zheng, Xiao-Yan Lu, Hai-Feng Qiao.

**Formal analysis:** Feng Xu, Xiao-Yan Lu, Hai-Feng Qiao, Ying Wang.

**Funding acquisition:** Yan-Li Zheng, Ying Wang.

**Investigation:** Yan-Li Zheng.

**Methodology:** Feng Xu, Xiao-Yan Lu, Hai-Feng Qiao.

**Project administration:** Feng Xu, Xiao-Yan Lu.

**Resources:** Hai-Feng Qiao, Ying Wang.

**Software:** Yan-Li Zheng.

**Supervision:** Xiao-Yan Lu.

**Validation:** Yan-Li Zheng, Hai-Feng Qiao.

**Visualization:** Feng Xu.

**Writing – original draft:** Feng Xu.

**Writing – review & editing:** Ying Wang.
